# Piloting a Clinical Decision Support Tool to Identify Patients With Social Needs and Provide Navigation Services and Referral to Community-Based Organizations: Protocol for a Randomized Controlled Trial

**DOI:** 10.2196/57316

**Published:** 2024-07-23

**Authors:** Elham Hatef, Thomas Richards, Kristin Topel, Sofia Hail, Christopher Kitchen, Katherine Shaw, Talan Zhang, Elyse C Lasser, Jonathan P Weiner

**Affiliations:** 1 Division of General Internal Medicine Department of Medicine Johns Hopkins School of Medicine Baltimore, MD United States; 2 Center for Population Health Information Technology Department of Health Policy and Management Johns Hopkins Bloomberg School of Public Health Baltimore, MD United States; 3 Division of General Pediatrics Department of Pediatrics Johns Hopkins School of Medicine Baltimore, MD United States; 4 Johns Hopkins Institute for Clinical and Translational Research Baltimore, MD United States; 5 Johns Hopkins Community Physicians East Baltimore Medical Center Baltimore, MD United States

**Keywords:** social needs, social determinants of health, clinical decision support tool, electronic health records, randomized controlled trial

## Abstract

**Background:**

Social needs and social determinants of health (SDOH) significantly outrank medical care when considering the impact on a person’s length and quality of life, resulting in poor health outcomes and worsening life expectancy. Integrating social needs and SDOH data along with clinical risk information within operational clinical decision support (CDS) systems built into electronic health records (EHRs) is an effective approach to addressing health-related social needs. To achieve this goal, applied research is needed to develop EHR-integrated CDS tools and closed-loop referral systems and implement and test them in the digital and clinical workflows at health care systems and collaborating community-based organizations (CBOs).

**Objective:**

This study aims to describe the protocol for a mixed methods study including a randomized controlled trial and a qualitative phase assessing the feasibility, acceptability, and effectiveness of an EHR-integrated digital platform to identify patients with social needs and provide navigation services and closed-loop referrals to CBOs to address their social needs.

**Methods:**

The randomized controlled trial will enroll and randomize adult patients living in socioeconomically challenged neighborhoods in Baltimore City receiving care at a single academic health care institution in the 3-month intervention (using the digital platform) or the 3-month control (standard-of-care assessment and addressing of social needs) arms (n=295 per arm). To evaluate the feasibility and acceptability of the digital platform and its impact on the clinical and digital workflow and patient care, we will conduct focus groups with the care teams in the health care system (eg, clinical providers, social workers, and care managers) and collaborating CBOs. The outcomes will be the acceptability, feasibility, and effectiveness of the CDS tool and closed-loop referral system.

**Results:**

This clinical trial opened to enrollment in June 2023 and will be completed in March 2025. Initial results are expected to be published in spring 2025. We will report feasibility outcome measures as weekly use rates of the digital platform. The acceptability outcome measure will be the provider’s and patient’s responses to the truthfulness of a statement indicating a willingness to use the platform in the future. Effectiveness will be measured by tracking a 3-month change in identified social needs and provided navigation services as well as clinical outcomes such as hospitalization and emergency department visits.

**Conclusions:**

The results of this investigation are expected to contribute to our understanding of the use of digital interventions and the implementation of such interventions in digital and clinical workflows to enhance the health care system and CBO ability related to social needs assessment and intervention. These results may inform the construction of a future multi-institutional trial designed to test the effectiveness of this intervention across different health care systems and care settings.

**Trial Registration:**

ClinicalTrials.gov NCT05574699; https://clinicaltrials.gov/study/NCT05574699

**International Registered Report Identifier (IRRID):**

DERR1-10.2196/57316

## Introduction

### Background

Social needs and social determinants of health (SDOH) significantly outrank medical care when considering the impact on a person’s length and quality of life [1]. Rising health care costs and worsening life expectancy in the United States are, in large part, the direct result of these unmet social needs and SDOH challenges [2-6]. Challenges related to social needs and SDOH disproportionately impact racial and ethnic minority populations and those with low income and are critical factors in explaining many health-related disparities, ultimately leading to higher mortality rates in these populations [7].

Currently, many sources of data on social needs and SDOH challenges can be found within a typical electronic health record (EHR) and some vendors have started adding specific data fields for collecting information on social needs and SDOH challenges. However, no universally accepted or standardized format exists for documenting social needs and SDOH information [8-13]. Furthermore, delivery systems can now take advantage of recent health IT advances in offering automated integration of new sources of relevant social needs and SDOH information [8,14,15]. The development of cross-provider regional health information exchanges, such as Maryland Health Information Exchange (known as CRISP), also offers the opportunity to connect social needs and SDOH data from multiple clinical organizations and to share information across medical and social sectors (eg, primary care practices and community-based organizations [CBOs]) [16].

The extensive range of available social needs and SDOH data will offer a better understanding of how individual and neighborhood challenges could impact a patient’s social needs and SDOH and increased risk of adverse health outcomes [17-22]. However, many technical and organizational challenges must be surmounted before social needs–integrated health IT solutions can be implemented on a wide scale. For example, the literature provides evidence of existing gaps in the development of best practices for integrating social needs and SDOH data into the clinical workflow and the clinicians’ core clinical decision support (CDS) tools [23] and data sharing with CBOs [24].

Also, multiple practical gaps exist in addressing social needs and SDOH in underlying care management tools. Over the past decade, the new generation of “value-based” health insurance plans and integrated delivery systems (eg, Patient-Centered Medical Homes, and Accountable Health Organizations) have fully embraced available Health IT systems to implement clinically oriented risk identification and stratification CDS tools addressing the needs of medically underserved patients [25]. These mechanisms are commonly used to identify and refer the subset of patients who are high-risk or high-cost (ie, with serious and multiple chronic conditions) who need care management services [25]. Today, more providers are placed at financial risk for a wider range of nontraditional performance-based target indicators (eg, neighborhood health indicators) and more diverse populations. Hence, there is a rapidly growing gap regarding the use of social needs and SDOH risk information, relative to clinical or morbidity risk factors, within current case finding and risk stratification methodologies. Such a gap is critical for the large proportion of patients, especially among minority populations, who, in addition to having high clinical needs, concomitantly face behavioral, socioeconomic, and community-resource challenges [26].

Some health plans and provider systems have started to address the social needs of their patients by implementing EHR-based survey assessment tools and navigation services in their clinical and digital workflows [27-31]. Moreover, a growing body of evidence such as the Accountable Health Communities model by the Centers for Medicare & Medicaid Services provides support for the implementation of such services in the health care settings and their impact on improving the care process and health outcomes [30,32,33]. However, automated and standardized social needs and SDOH risk identification metrics and data collection and curation processes are sorely lacking. To avoid burdensome and inefficient social needs assessment (data collection from all patients at each visit), it will be essential to develop automated screening tools using EHR or community databases to help identify the subset of patients who would most benefit from EHR-integrated social needs assessment and data collection [34]. Also, currently in most health systems, the referral to CBOs is based on clinic staff’s one-off attempt to identify available CBOs and to make referral arrangements. And, in cases where an automated directory of services is used, information is rarely exchanged that might benefit the patient [24,29,30]. The disconnect between health systems and CBOs and the lack of established data-sharing mechanisms have many negative implications for the patient and the system. For example, it is difficult for the providers to identify whether the social needs of their patients were addressed [8,28,35]. From a system perspective, such disconnect makes it difficult to evaluate the impact of identification, assessment, and referral of social needs on health outcomes and health care use, as well as to evaluate the efforts of health systems to address disparate access to medical and social services [28].

Thus, integrating data on social needs and SDOH challenges along with clinical risk information within operational CDS systems built into EHRs is an effective approach to addressing health-related social needs. To achieve this goal applied research is needed to (1) identify optimal solutions for the effective collection and application of social needs and SDOH information within EHRs; (2) develop EHR-integrated CDS tools and closed-loop referral systems; and (3) implement the CDS tools and closed-loop referral systems in the digital and clinical workflows at health care systems and collaborating CBOs.

### Primary Objective

The objective of this mixed methods study including a randomized controlled trial (RCT) and a qualitative phase is to address the current gaps in the EHR-based social needs assessment and navigation services. Thus, we plan to assess the feasibility, acceptability, and effectiveness of an EHR-integrated digital platform to identify patients with social needs and provide navigation services including CBO referrals. The digital platform includes a CDS tool and a closed-loop CBO referral. The CDS tool contains a social risk score and an overview of different risk factors and comorbidities contributing to the patient’s social risk score. The CDS tool also provides recommendations on assessment for social needs based on the social risk score and other available information (refer to Multimedia Appendix 1 for an explanation and overview of the CDS tool).

Using both patient- and population-level data, we developed a social predictive risk score based entirely on electronic information readily available within most health care delivery systems. We also validated the model using multiple denominators to ensure generalizability and retrained and tested the model for each subpopulation of interest (eg, individuals aged 65 years or older, racial and ethnic minority populations, and those living in the most and least disadvantaged neighborhoods) [34]. The predictive risk score helps providers systematically identify patients at risk of having social needs based on their demographic characteristics, clinical comorbidities, previous social needs, and clinical outcomes such as hospitalization and emergency department visits. Those patients represent likely targets for further assessment of their social needs and ultimately potential referral to CBOs to address such needs. Using this systematic electronic case-finding screening approach helps the health care system avoid burdensome and inefficient social needs assessment (eg, primary data collection from every patient at every visit).

### Hypothesis

Building on the current evidence on the experience of patients, providers, and health care systems with the EHR-based social needs assessment and navigation services [32] as well as the impact of such systems on care process and health outcomes [33], we plan to test the central hypothesis that implementing the digital platform for social needs identification and providing navigation services will (1) be feasible in digital and clinical workflows of primary care ambulatory clinics and CBOs; (2) be acceptable by patients, clinical providers, and CBO staff; and (3) demonstrate effectiveness in improving care process and ultimately the clinical outcomes.

## Methods

### Study Overview and Design

**Figure 1:**
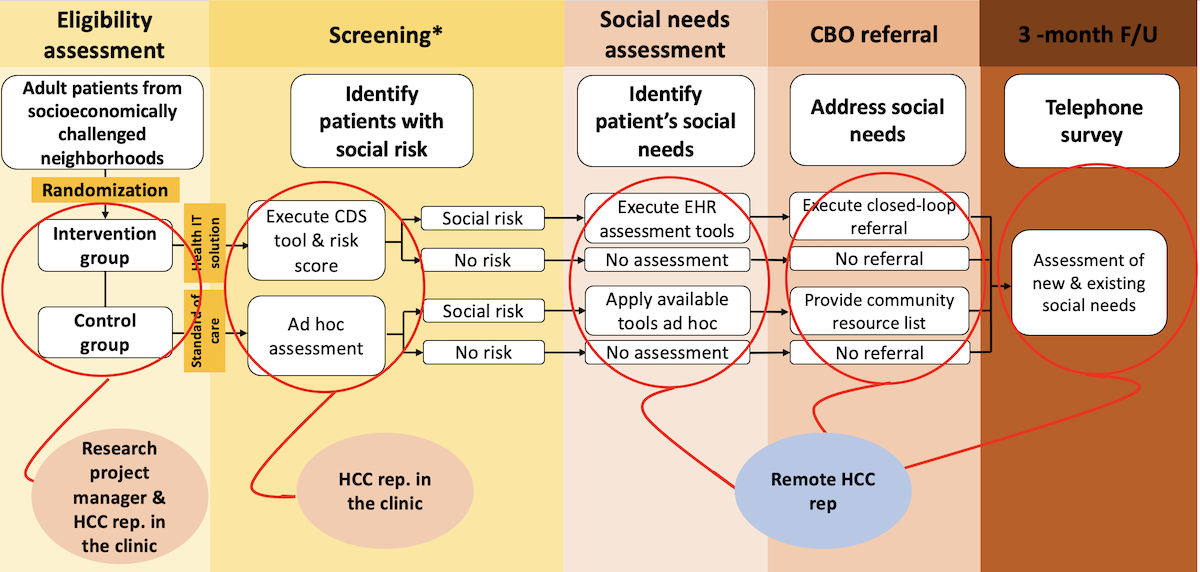
An overview of a randomized controlled clinical trial to identify patients with social needs and provide navigation services. *In the screening phase, the HCC team (a team of trained social workers and care managers, serving as a hub at Johns Hopkins Health System providing standardized social needs assessment and navigation services) uses the social risk score available in the CDS tool for the intervention arm to decide the social risk of the patient and the need for further assessment. CBO: community-based organization; CDS: clinical decision support; EHR: electronic health record; F/U: follow-up; HCC: Hopkins Community Connection.

We will apply an RCT design to compare selected outcomes between the intervention (digital platform) and control (standard of care) groups during their primary care ambulatory visits. The RCT will take place in 5 primary care ambulatory clinics at Johns Hopkins Health System (JHHS) and 5 CBOs in Baltimore City. The CBOs are specialized in providing services related to different categories of social needs including residential instability (ie, homelessness, housing insecurity, and poor-quality housing), food insecurity, and transportation. We will identify a clinical provider champion in each pilot primary care clinic who will serve as the liaison between the study team and the clinic. The provider champion will help the research team with the implementation of the platform in the digital and clinical workflow and will advocate for the study among other providers. Figure 1 describes the RCT’s procedural flow.

We will identify eligible patients via EHR in the JHHS pilot primary care clinics. A research project manager will contact patients after obtaining their permission via mail, email, and patient portal communications. After verifying eligibility and introducing the study, interested patients will complete consent processes. We will randomize the patients to the intervention or control arm for a 3-month study period. The patients in the intervention group will be contacted by staff at Hopkins Community Connection (HCC; a team of trained social workers and care managers, serving as a hub at JHHS providing standardized social needs assessment and navigation services) within 48 weekday hours of enrollment and receive social needs assessment and navigation services based on their social risk score and other information available in the CDS tool. The patients in the control group will receive the standard of care consisting of provider assessment, assessing, and addressing social needs on an as-needed basis based on the information provided during the visit. The patients in the intervention group will complete an exit interview via phone 3 months after the intervention, assessing their experience with the digital and clinical work process. For patients in the control group, we will retrieve any information on social needs assessment and referral services provided as a standard of care from their EHR.

To evaluate the feasibility and acceptability of the digital platform and its impact on the clinical and digital workflow and patient care we will conduct focus groups with the care teams at various pilot primary care clinics (eg, clinical providers), HCC staff, and CBO staff to determine satisfaction of users and identify any facilitators or barriers to using the digital platform.

### Randomized Controlled Trial

#### Patient Enrollment—Identifying Eligible Patients

To identify eligible patients, we will use the following inclusion or exclusion criteria: adult (18+ years of age) patients living in socioeconomically challenged neighborhoods. First-generation immigrants who may be non–English speakers will be eligible for enrollment. The JHHS-EHR does not contain data on patients’ income. Therefore, we will consider enrollment in Medicaid and living in a neighborhood with a high poverty level and socioeconomic challenges as proxies for low income. We will use the patient’s address in the EHR to identify his or her neighborhood of residence and use the area deprivation index [36], a population-level composite measure ranking the neighborhoods based on their poverty level, using data from the US Census Bureau American Community Survey. We will consider area deprivation index at the 75th percentile as a proxy for the most deprived neighborhoods. We will consider children, individuals with high levels of income, and patients who already have an HCC care team member assigned to them with a start date within the last 90 days and no end date as noneligible to enroll in the study.

Every week, our data analyst will use the abovementioned inclusion or exclusion criteria to identify eligible patients with a primary care appointment in 3 weeks in one of the pilot primary care clinics at JHHS. The data analyst also will update the list of eligible patients to add patients with a newly scheduled appointment within 3 weeks of their appointment. We will use the JHHS-EHR patient portal (EPIC MyChart) to send automated messages to eligible patients, inform them about the study, and provide background information on the project. If patients agree to participate in the study, the research project manager will contact them to complete the consent process. The research project manager will also reach out to patients via email and paper mail to ask permission to contact them by phone. The research project manager will contact patients who will not opt out of future communications with the study team to inform them of the study, provide information, and obtain consent. We will also distribute flyers, containing a 1-page graphic document summarizing the study process, in the pilot primary care clinics to inform patients of the ongoing study and provide contact information for them to get connected to the research project manager (refer to Multimedia Appendix 2).

Moreover, we will contact the providers via the EHR secure messaging before an eligible patient visits and inform them about the patient’s eligibility for our study. The providers inform the patients about the study and if the patients agree to participate, they will communicate this information with the research project manager via secure messaging. This process will be completed using an EHR SmartPhrase to populate a prefilled form containing a 1-page summary of the project along with the contact information of the research project manager and add it to the visit summary. This document will be printed at the clinic or sent via email or patient portal to the patient.

#### Patient Enrollment—Consenting and Randomization

We will use both teleconsent via DocuSign and in-person consenting in the clinic when possible to obtain informed consent from eligible patients. We will use the DocuSign 21 CFR Part 11-compliant software (DocuSign, Inc) to obtain a secure electronic signature [37]. The consent discussion will take place via phone or videoconference (eg, Zoom; Zoom Video Communications) and patients will be given adequate time to consider the research study and ask questions before signing the consent form. For patients unable to use the DocuSign to complete the consent process the HCC staff will approach the patients in the clinic on the day of their visit and obtain the consent after providing information about the study, answering their questions, and giving them adequate time to read through the consent form.

We will use stratified blocked sampling through REDCap (Research Electronic Data Capture) with each pilot primary care clinic serving as a stratum to generate the randomization list. Each pilot primary care clinic will have blocks of different sizes (eg, 4, 6, and 8), which will be randomly distributed (eg, one clinic may have blocks of 4,6, 8, 6, 4, 8, …. and another one may have blocks of 6,8, 4, 4, 8, 6, …). Each block will contain an equal number of intervention and control assignments with different orders. This strategy helps ensure an equal number of patients in the 2 arms of the study at any time point during the enrollment and ensures complete masking of the randomization process, with no chance to predict the next randomization assignment in a clinic. The randomization will be completed for each patient after they complete the consent process. On the day of an eligible patient’s visit, the research project manager will monitor the patient’s arrival in the clinic through EHR, and when the patient gets to the clinic, they will use the REDCap randomization list to receive the randomization assignment for each patient and to record the patient’s name and ID with their assigned group (ie, intervention or control arm). They will communicate the randomization assignment with the HCC staff in the clinic through EHR secure messaging.

#### Planned Intervention

Once consented to and randomized into the intervention or the control arms, the patients will be seen at their regularly scheduled appointment in the primary care clinic.

##### Intervention Arm

For patients in the intervention arm the HCC staff in each clinic will use the CDS tool to review their social risk score. If the patient is identified as with medium to high social risk based on their risk score and other information provided in the CDS tool, the HCC staff will perform further assessment of their social needs using available standard social needs assessment tools in the EHR [38,39]. For patients with low risk, the HCC staff will review the social needs history and other information depicted in the tool and decide whether to perform further assessment. A summary sheet will be provided to the HCC staff as a guide to all the details depicted in the CDS tool to help with their decision-making (refer to Multimedia Appendix 1). If the HCC staff decides to screen the patient for social needs, they will leave a secure message for the clinical provider in the EHR, informing them about the risk score and the decision regarding the assessment. The clinical provider will have the option of checking the risk score on the CDS tool, the summary of patients’ previous social needs, and other factors contributing to the social risk score.

To perform further assessment of the social needs the HCC staff will reach out to the patients over the phone and will perform the assessment. If any social needs are identified and the patient agrees to address those needs the HCC staff will work with the patient to set goals for different identified social needs and provide navigation services including CBO closed-loop referral to achieve those goals. The HCC staff will use FindHelp [40], a Health Insurance Portability and Accountability Act–compliant database and referral platform for social needs assessment, documentation, and referral to CBOs. FindHelp also provides the opportunity for real-time communications between clinical providers and CBOs.

After an assessment of the social needs and providing available navigation services at JHHS the HCC staff will generate a summary report through FindHelp and alert the CBOs about the referred patients. CBOs will have access to FindHelp and can review patients’ social needs assessment and summary reports. After meeting with the referred patient and identifying resources to address their social needs, CBOs generate a summary report, which will be accessible to the HCC staff and clinical provider teams.

##### Control Arm

For patients in the control arm the standard-of-care assessment and addressing of social needs will be provided. The assessment will not be automated with precollected information as is designed in our CDS tool. The assessment will be on an ad hoc basis, which may include completing currently available social needs assessment tools in the clinics and providing information to the patients about available community-based resources.

##### Follow-up

For patients in the intervention arm of the study who get screened by the HCC, after their case has been closed (approximately 3 months after enrollment), the HCC staff will conduct a telephone survey with the patients to assess any changes in their social needs, the degree of which the social needs were met, and patient’s satisfaction with the process for social needs assessment and navigation services as well as the services they received. For patients in the control group, we will retrieve any information on social needs assessment and referral services provided as a standard of care from their EHR. Moreover, for all patients in the study, we will conduct a secondary data analysis to assess the impact of the CDS tool and navigation services on selected clinical outcomes such as hospitalization and emergency department visits (refer to Multimedia Appendix 3 for an overview of the clinic workflow).

#### Sample Size

We plan to enroll approximately 295 patients in each study arm. The sample size calculation is based on addressing the primary outcome of the study namely the effectiveness of the proposed digital process and its impact on process measures. The primary outcome is defined as the change in the number of social needs identified during the visit at the 3-month follow-up telephone survey versus the baseline visit comparing the intervention and control arms. Such a sample will provide 80% power in 2-sided tests with a type I error rate of 5% to detect standardized small effect sizes (Cohen *d*=0.17-0.20 as is estimated in similar studies of social needs assessment and navigation services [30]). Sample size estimates conservatively account for a 20% loss to follow-up.

#### Data Collection and Measures

We will collect demographic data (ie, age, gender, race, insurance type, language preference, and need for interpreter), clinical comorbidities (using the Johns Hopkins Adjusted Clinical Groups [41], one of the world’s most widely used population-based predictive modeling and case-finding methodologies), previous social needs, and health outcomes (ie, hospitalization and emergency department visits) from the JHHS-EHR. The information related to the assessment for social needs, and navigation services including referral to CBOs, and their 3-month follow-up interview will be documented in the FindHelp platform [40]. We will also obtain information on the randomization assignment for each patient through REDCap and collect the information on the social risk score at the time of randomization from our CDS tool. The data generated through the CDS tool will be stored in the Health Insurance Portability and Accountability Act–compliant JHHS enterprise data warehouse.

### Qualitative Assessment—Focus Groups

#### Participant Enrollment

We will evaluate the EHR-integrated digital platform (the CDS tool and the CBO closed-loop referral) from the health system and community organization perspectives. Thus, we will conduct focus groups with provider teams such as physicians, nurses, social workers, care managers, community program coordinators or patient navigators in the clinics and at HCC, and staff of CBOs to determine the satisfaction of the users with the digital platform, determine whether there are any facilitators or barriers to using the platform, and generate ideas on who to improve the platform and the digital process. The research project manager will identify individuals who interacted with the platform in each pilot clinic, HCC, and CBOs and invite them to join the focus groups. We will work with the provider champion at each clinic site to inform other provider teams about the focus groups and invite them to the study. We will also work with the HCC program manager and the CBO staff to identify qualified individuals for the focus groups. We will use a convenience sampling method to select and enroll qualified individuals.

#### Conducting Focus Groups

The focus groups will be 90 minutes each. Participants will be invited via email to join a focus group at a convenient date and time for their schedule and will consent to this part of the study on the day of the meeting. The email will provide a summary of the study and the purpose of the focus group; the date, time, and duration of the focus group; and the contact information of the study principal investigator (PI) to answer any questions of the potential participants before they agree to participate. The focus groups will be conducted under the supervision of the study PIs and study coinvestigators with expertise in qualitative research. Participants in the focus groups will be asked a variety of questions on their interaction with the digital platform, their perception of how it fits within the clinic and CBO digital and clinical workflow, their satisfaction with the platform, and how it can be improved. Focus groups will be recorded in Zoom and transcribed using the Zoom-automated service. We will review the transcripts to address any inconsistency in the automated Zoom transcripts, deidentify, and upload them into MAXQDA (VERBI Software) [42] for data management and analyses.

#### Sample Size

We aim to enroll and engage a variety of clinical providers, HCC, and CBO staff. We will attempt to conduct six 90-minute meetings for 6 months to collect input on the potential challenges in the implementation and use of the digital platform. We plan to have 6-10 individuals at each meeting for a total of 36-60 individuals from a variety of positions within the health system and CBOs.

### Outcome Assessment

We will assess whether the EHR-integrated digital platform is superior to the standard of care and more feasible and acceptable. Thus, we will track the outcomes of feasibility, acceptability, and effectiveness through the RCT and qualitative study components (Table 1). We will report feasibility outcome measures as weekly use rates of the digital platform (the CDS tool and closed-loop CBO referral). The acceptability outcome measure will be the provider’s responses to the truthfulness of a statement indicating a willingness to use the digital platform in the future. Effectiveness (ie, the primary outcomes) will be measured by tracking the 3-month process and clinical outcomes versus the baseline visits comparing the intervention and control arms.

**Table 1:** Outcome measures for digitally enabled social needs assessment and intervention.

Selected measure		Data source and collection method	Timing
**Outcome category: feasibility**			
	Use of the EHR^a^-integrated digital platform—quantitative measures—including weekly rates of log into the CDS^b^ tool and closed-loop CBO^c^ referral	JHHS^d^-EDW^e^ FindHelp Data	Enrollment to 3-month follow-up
	Types of use—qualitative measures—including checking the risk score and reviewing the summary report of contributing factors to social risk	Focus group transcript	During and after completion of the RCT^f^
	Types of use—quantitative and qualitative measures—including performing social needs assessment, setting goals for the patients and follow-up with patients and CBOs, and performing follow-up interviews	JHHS-EDW, FindHelp data, and focus group transcript	During and after completion of the RCT
**Outcome category: acceptability**			
	Willingness to reuse—quantitative and qualitative measures	Provider focus group transcript	During and after completion of the RCT
	Willingness to use the EHR-integrated digital platform again	Patient follow-up interview	3-month follow-up
**Outcome category: effectiveness**			
	Impact on process measures—quantitative measures—including change in the number of identified social needs from enrollment to follow-up, comparing intervention and control arms, and change in the number of navigation services and CBO closed-loop referrals from enrollment to follow-up, comparing intervention and control arms	JHHS-EDW and FindHelp data	Enrollment and 3-month follow-up
	Impact on clinical outcomes—quantitative measures—including change in the hospitalization rates from enrollment to follow-up, comparing intervention and control arms, and change in the emergency department visit rates from enrollment to follow-up, comparing intervention and control arms	JHHS-EDW	Enrollment and 3-month follow-up

^a^EHR: electronic health record.

^b^CDS: clinical decision support.

^c^CBO: community-based organizations.

^d^JHHS: Johns Hopkins Health System.

^e^EDW: enterprise data warehouse.

^f^RCT: randomized controlled trial.

### Analysis Plan

We will adhere to the standards for trial design, analysis, and reporting per the CONSORT (Consolidated Standards of Reporting Trials) [43]. We will perform univariate analysis including demographic characteristics, clinical comorbidities, previous social needs, and clinical outcomes such as hospitalization and emergency department visits among all patients as well as in the intervention and control arms. We will use frequencies, means, and standard deviations related to feasibility and acceptability outcomes. For the effectiveness outcomes, we will use the change in the number of identified social needs and provided navigation services from baseline to 3-month follow-up and proportion with any hospitalization and emergency department visits comparing the 2 study arms using *t* test and Pearson chi-square tests. We will also use a logistic regression model to include other potential predictors of the outcome to improve the precision of the estimate. All trial procedures will also be in concordance with ClinicalTrials.gov regulations (NCT05574699).

For focus groups, we will deidentify the transcripts and upload them into MAXQDA [42] for data management and analyses. We will deductively develop an initial codebook from the interview guide and apply the deductive codes to the first few interview transcripts. In addition, we will inductively identify emergent subcodes for each parent code, resulting in several subcodes. This initial coding process will be conducted by the PI and coinvestigators with expertise in qualitative research. We will discuss the disagreements in coding and reach a consensus by mutual agreement. One coder will then code the remainder of the interview data and both reviewers will discuss emergent findings and modifications to the coding framework. We will engage in a process of constant comparison of emergent findings throughout the analytic process, and when no new codes can be identified, we will consider thematic saturation to have been achieved.

### Ethical Considerations

This study protocol was approved by the Johns Hopkins University, School of Medicine’s institutional review board (IRB; #IRB00354803).

#### Data Management Plan

We will convene a data and safety monitoring board (DSMB) to safeguard the interests of study patients, ensure safety and adherence to human subjects’ protection policies, and monitor the overall conduct of the study. The DSMB will be an independent advisory group to the project PI. Communication with DSMB members will be primarily through the PI. It is expected that other study investigators will not communicate with DSMB members about the study directly, except when making presentations or responding to questions at DSMB meetings or during conference calls. The DSMB will meet twice annually throughout the study, when the DSMB will review the progress of the study (accrual rate, protocol deviations, and interim study analyses, if recommended) and make recommendations.

#### Privacy and Confidentiality

To safeguard participant information data will be deidentified for analysis and reporting of the results.

#### Compensation Details

Patients who participate in the RCT will receive services based on their social needs. After completion of the 3-month follow-up interview, patients will receive a $20 gift card for their participation in the study. There are no monetary tokens or compensation directly to the participants in the focus groups.

#### Plan for Reporting Unanticipated Problems or Study Deviations

It is anticipated that patients will experience more benefits than risks from their participation in the trial. There are only minimal risks associated with this study including the risk of unintended consequences, such as social risk profiling that could lead to bias and stereotyping in the delivery of care to patients. To address this risk, the study team will incorporate patients’ preferences into the design and use of the EHR-integrated digital platform and will provide training for the clinic, HCC, and CBO staff on how to use the information available in the CDS tool in their communications with patients. There might also be some ethical concerns about identifying social needs beyond the major categories we have specified in this study (ie, residential instability, food insecurity, and transportation) without addressing them. We refer to such events as alerts and have well-developed procedures for their management. All alerts are reported to the DSMB on a biannual basis. However, the reporting of alerts to the IRB is not required. Details about specific alerts and actions taken are shown in Table 2.

If a serious adverse event happens the DSMB will be notified by the PI within 48 hours of initial notification to the project team. All members of the DSMB will receive copies of all safety reports at the time of submission to the IRB. In addition, a listing of all adverse events and their attribution (eg, study-related, intervention-related, or unrelated to study or treatment) will be provided to the DSMB monthly.

**Table 2:** Specific alerts and actions taken for reporting unanticipated problems or study deviations in the randomized controlled trial to identify patients with social needs and provide navigation services.

Alert	Action taken
Suicidal ideation, threats to hurt self	If an HCC^a^ staff encounters a situation in which the person threatens to hurt themself immediately, then the physician at the clinic site is informed and the person is referred to the emergency department for observation and psychiatry consult. If the person is not an immediate threat to self, then the physician at the clinic site is informed and the person is actively encouraged to make a psychiatry appointment and the contact is made for the person if they choose. The HCC staff also informs the person that a member of the team will be contacting him or her shortly to follow up. Immediately after the visit, the PI^b^ will be notified of the situation. The PI will complete the alert form and give the form to the study project manager for filing.
Evidence of abuse and intimate partner violence	Evidence of physical abuse is as follows: (1) patient states to the HCC staff that abuse occurs and (2) the HCC staff observes physical evidence (eg, black eye, black and blue marks on arms or legs). The HCC staff informs the physician at the clinic site. The physician obtains further information from the patient and will contact Adult Protective Services. Immediately after the visit, the PI will be notified of the situation. The PI will complete the alert form and give the form to the study project manager for filing. Note: The possibility of informing an agency about an abusive situation is stated in the informed consent.

^a^HCC: Hopkins Community Connection.

^b^PI: principal investigator.

### Dissemination Plan

We will disseminate the study results regardless of effect direction and size through publications in peer-reviewed journals and presentations at conferences. Moreover, we will develop guidelines and algorithms to support other health care systems wishing to apply our methods and tools. These will include logic flow for an EHR-integrated digital platform containing the risk assessment process, the navigation services, and the referral process. In addition, we will provide information on challenges surrounding data sharing between primary care practice sites and CBOs. Our recommendations will suggest how to address the variabilities in information technology infrastructure across different CBOs. We will also develop recommendations for how best to scale the implementation of the EHR-integrated digital platform, across the entire JHHS, regionally, and nationally. Finally, we will vigorously disseminate our findings and methods to major provider networks, technology vendors, policy makers, and other stakeholders.

## Results

We started enrollment in June 2023 and will complete it in January 2025. The analysis is expected to be completed by March 2025 with results published in spring of 2025. We plan to perform an interim analysis when the enrollment reaches 50% of the planned sample size.

## Discussion

This study will provide evidence on the best approaches to create and implement a digital workflow for social needs assessment and navigation services and assess whether the implementation of the EHR-integrated digital platform and process is feasible in a health care system and in CBOs, acceptable among the frontline providers and CBOs staff, and effective in impacting health-related outcomes. If the planned intervention demonstrates greater improvements in 1 or more study outcomes, these findings can be tested and spread through future implementation research and processes. We acknowledge that the inadequate available resources in the community may limit the ability of the CBOs to address identified social needs, which may impact the effectiveness of the digital platform.

Importantly, the application of mixed methodology and the addition of focus groups with clinical and social stakeholders are unique aspects of this study. This approach will help identify barriers and facilitators to future implementation of the digital platform and potential needed modifications to the digital platform and workflow to ensure the sustainable use of the platform in clinical settings. Such modifications will facilitate future uptake of the digital platform should it prove effective. Our methodology can be used to examine the effects of similar EHR-integrated digital platforms to address unmet social needs in the health care systems and collaboration with community-based organizations.

## Appendix

Multimedia Appendix 1 Randomized controlled clinical trial to identify patients with social needs and provide navigation services—clinical decision support tool explanation sheet.

Multimedia Appendix 2 Randomized controlled clinical trial to identify patients with social needs and provide navigation services—clinic flyer.

Multimedia Appendix 3 Randomized controlled trial to identify patients with social needs and provide navigation services—clinic workflow.

## Abbreviations

CBO: community-based organization
CDS: clinical decision support
CONSORT: Consolidated Standards of Reporting Trials
DSMB: data and safety monitoring board
EHR: electronic health record
HCC: Hopkins Community Connection
IRB: institutional review board
JHHS: Johns Hopkins Health System
PI: principal investigator
RCT: randomized controlled trial
REDCap: Research Electronic Data Capture
SDOH: social determinants of health
