# Supplementary material for: Piloting a Clinical Decision Support Tool to Identify Patients With Social Needs and Provide Navigation Services and Referral to Community-Based Organizations: Protocol for a Randomized Controlled Trial
Source: JMIR Res Protoc. 2024 Jul 23;13:e57316. doi: 10.2196/57316 (PMC11303893; doi:10.2196/57316)
Supplement: Multimedia Appendix 1 [file resprot_v13i1e57316_app1.docx]

What Is The Social Risk Score and How Can a Provider Utilize It?

The Johns Hopkins Center for Population Health IT (CPHIT) has developed a multi-level social risk score to integrate available digital information on social needs and social determinants of health (SDOH) in the structured electronic health record (EHR) to identify patients at risk of having social needs. The information in the providers’ free-text notes is not included in the calculation of the social risk score.

This risk score along with relevant details has been summarized into a clinical decision support (CDS) tool that can be accessed by providers through Johns Hopkins Health System (JHHS) EHR. The CDS tool provides the opportunity for the provider teams to identify patients at risk of having high social needs, for an in-depth assessment of social needs and referral to community-based organizations where their needs can be addressed. The CDS tool enables the delivery of longitudinal, actionable summaries of evidence about a patient's current social risk and different factors that contributed to their current risk.

Providers will see the following after opening the CDS tool:

1. **The Social Risk Score**

The first section shows the current social risk score prediction based on the latest calculation date. The risk score ranges from 0-100% and is categorized into low (0-33%), medium (34%-66%), and high (67%-100%).

The CDS tool provides recommendations to the clinical providers about the risk score; patients with **medium** or **high** social risk scores are required to complete further assessment of their social needs and receive navigation services including referral to community-based organizations based on the specific identified social needs. For patients with **low** risk, further assessment should be completed in instances where other details of the CDS tool suggest a potential high risk of having social needs.

The images below show two examples of the current social risk scores for patients with low and medium risk.


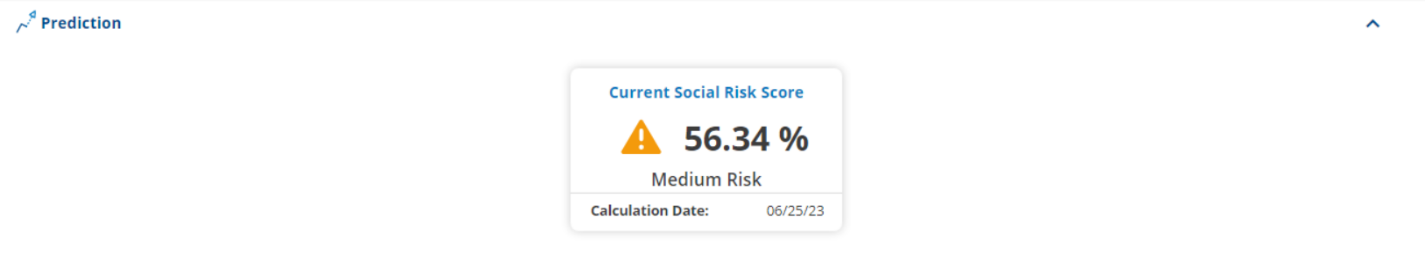

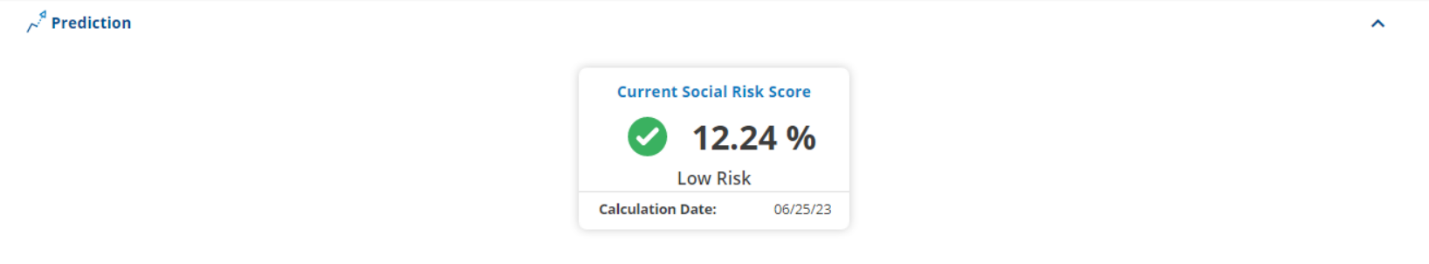


**Figure 1. Two Examples of the CDS Tool Presenting Current Social Risk Score for Patients with Low (Left) and Medium (Right) Risk.**

1. **Patient Events**

The CDS tool also captures emergency department (ED) visits and hospital admissions in the past two years, reporting these events in 3-month intervals. Each icon represents a specific event and the exact date of the event can be seen by selecting the icon.


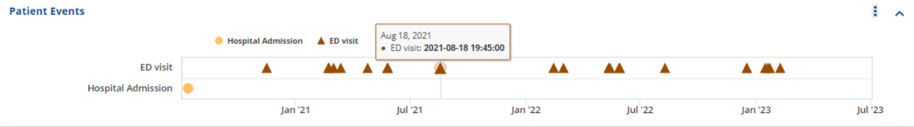


**Figure 2. Example of the CDS Tool Presenting Information on ED Visits and Hospital Admissions in the Past Two Years, Reporting Events in 3-month Intervals.**

1. **Morbidity/Resource Use (RUB)**

The CDS tool contains the Johns Hopkins *Adjusted Clinical Groups’ (ACG®) Resource Utilization Bands (RUBs),* presenting the RUB values over the past two years*.* ACG® is one of the world's most widely used population-based predictive modeling/case-finding methodologies. RUBs are a simplified ranking system of overall morbidity levels. Individuals expected to use the same level of resources, regardless of the type of illness, are grouped together.


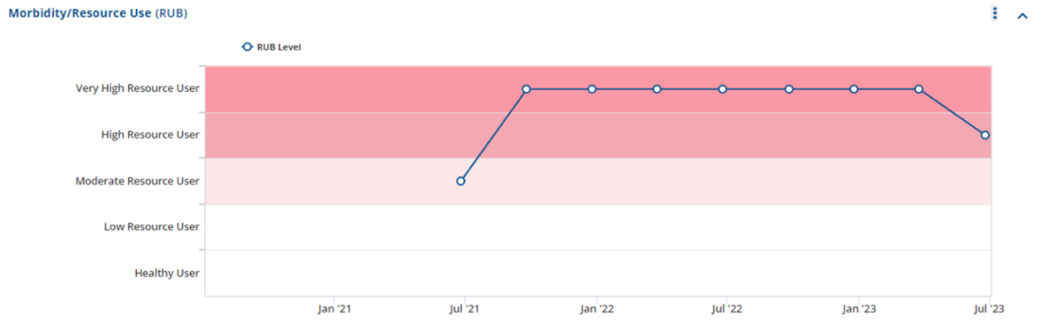


**Figure 3. Example of the CDS Tool Presenting Information on RUBs in the Past Two Years, Reporting Events in 3-month Intervals.**

1. **The Longitudinal View of the Social Risk Score**

The CDS tool presents a longitudinal view of the social risk score in the past two years with 3-month intervals. By selecting a specific time point, providers can see which factors contributed to the changes in the risk score over time.


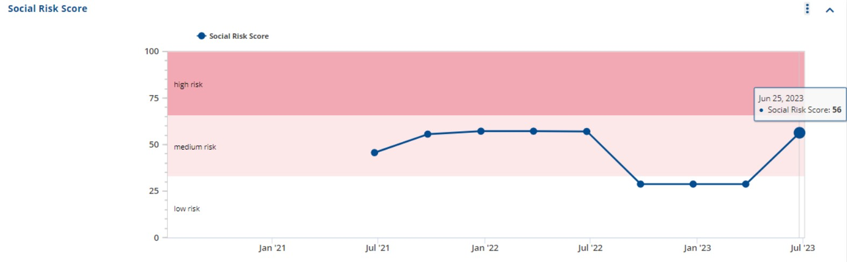


**Figure 4. Example of the CDS Tool Presenting a Longitudinal View of the Social Risk Score in the Past Two Years, Reporting Events in 3-month Intervals.**

1. **Previous Social Needs**


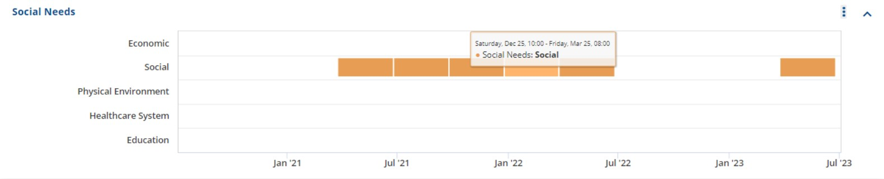
Lastly, the CDS tool shows specific social needs captured in the past two years in the EHR structured data through the use of ICD-10 codes and Wellness Registry flowsheets that generate the EPIC-SDOH wheel. We categorized documented social needs into five domains: Economic, Social, Physical Environment, Healthcare system, and Education, presented on the Y-axis, and 13 sub-domains, presented in the boxes on different rows. Selecting an item will show what specific subdomains of social needs were recorded and the details of where the data came from (ICD-10 codes or Wellness Registry which collect data from different sources in EPIC).


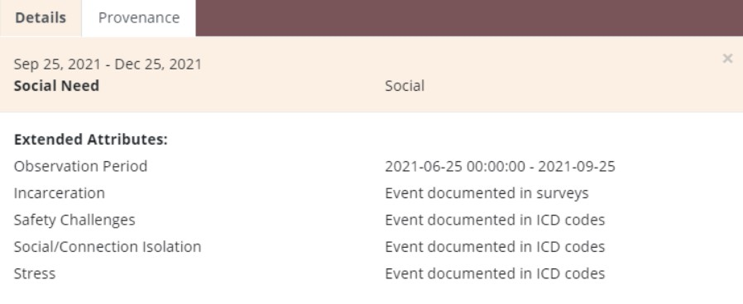


**Figure 5. Example of the CDS Tool Presenting Specific Social Needs Captured in the Past Two Years, Reporting Events in 3-month Intervals (Top) and the Details of Data Sources for Documented Social Needs (Bottom).**
