# Supplementary material for: Piloting a Clinical Decision Support Tool to Identify Patients With Social Needs and Provide Navigation Services and Referral to Community-Based Organizations: Protocol for a Randomized Controlled Trial
Source: JMIR Res Protoc. 2024 Jul 23;13:e57316. doi: 10.2196/57316 (PMC11303893; doi:10.2196/57316)

**Participants needed for a research program about social needs such as food problems, housing issues, and lack of transportation services to attend medical appointments**


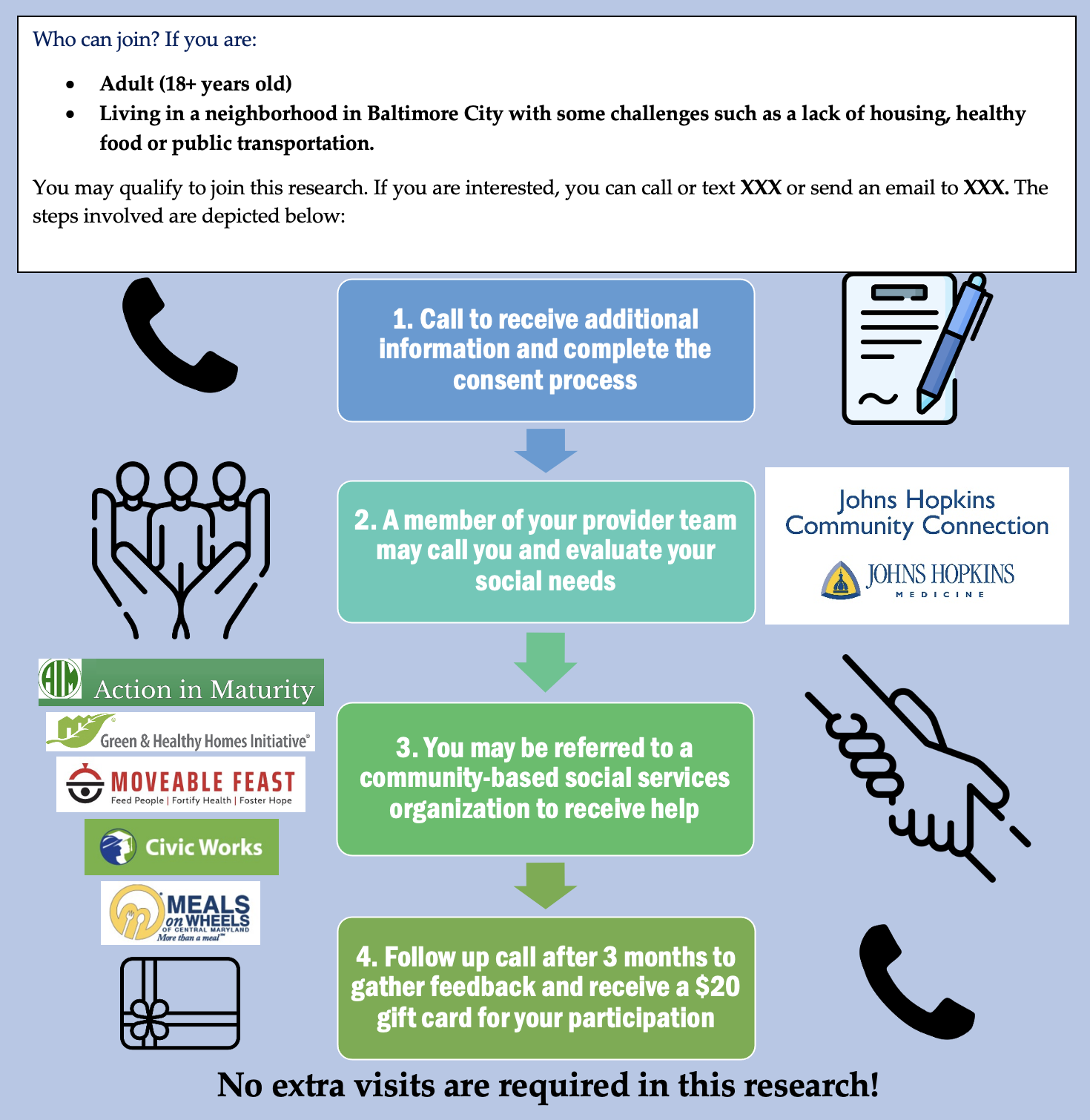

Supplement: Multimedia Appendix 2 [file resprot_v13i1e57316_app2.docx]
