# Supplementary material for: Piloting a Clinical Decision Support Tool to Identify Patients With Social Needs and Provide Navigation Services and Referral to Community-Based Organizations: Protocol for a Randomized Controlled Trial
Source: JMIR Res Protoc. 2024 Jul 23;13:e57316. doi: 10.2196/57316 (PMC11303893; doi:10.2196/57316)
Supplement: Multimedia Appendix 3 [file resprot_v13i1e57316_app3.docx]

## **Randomized Controlled Trial to Identify Patients with Social Needs and Provide Navigation Services – Clinic Workflow**


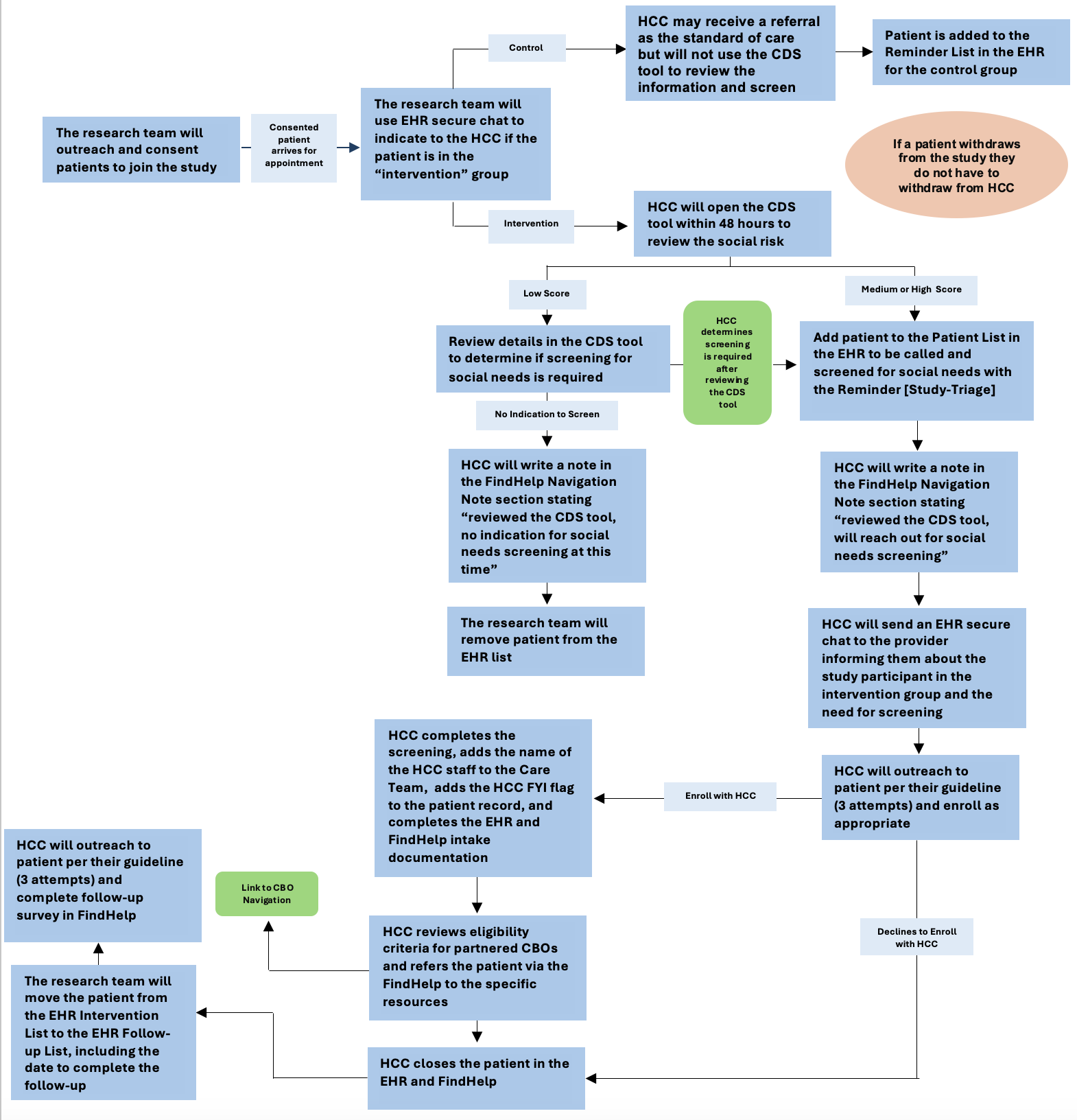


The HCC is a team of trained social workers and care managers, serving as a hub at JHHS providing standardized social needs screening and navigation services. CBO: Community-Based Organization, CDS: Clinical Decision Support, HCC: Hopkins Community Connection, EHR: Electronic Health Record, JHHS: Johns Hopkins Health System.
